# Dietary resistant starch dose-dependently reduces adiposity in obesity-prone and obesity-resistant male rats

**DOI:** 10.1186/1743-7075-9-93

**Published:** 2012-10-25

**Authors:** Damien P Belobrajdic, Roger A King, Claus T Christophersen, Anthony R Bird

**Affiliations:** 1 Commonwealth Scientific & Industrial Research Organisation (CSIRO) Food Futures Flagship, Adelaide, Australia; 2 CSIRO Animal Food and Health Sciences, Adelaide, Australia

**Keywords:** Resistant starch, Adiposity, Incretin, Short chain fatty acid, Insulin sensitivity

## Abstract

**Background:**

Animal studies show that diets containing resistant starch (RS) at levels not achievable in the human diet result in lower body weight and/or adiposity in rodents. We aimed to determine whether RS dose-dependently reduces adiposity in obesity-prone (OP) and obesity-resistant (OR) rats.

**Methods:**

Male Sprague–Dawley rats (*n*=120) were fed a moderate-fat, high-energy diet for 4 wk. Rats that gained the most weight (40%) were classified as obesity-prone (OP) and obesity-resistant (OR) rats were the 40% that gained the least weight. OP and OR rats were randomly allocated to one of six groups (n=8 for each phenotype). One group was killed for baseline measurements, the other five groups were allocated to AIN-93 based diets that contained 0, 4, 8, 12 and 16% RS (as high amylose maize starch) for 4 wk. These diets were matched for total carbohydrate content. At 0, 4 and 7 wk from the start of the study insulin sensitivity was calculated by homeostasis model assessment of insulin resistance (HOMA-IR) and adiposity was determined by dual-energy X-ray absorptiometry (DXA). At 8 wk, rats were euthanized and fat pad weights, intestinal digesta short chain fatty acid (SCFA) pools and plasma gut hormone levels were determined.

**Results:**

Obesity prone rats gained less weight with 4, 12 and 16% RS compared to 0% RS, but the effect in OR animals was significant only at 16% RS. Irrespective of phenotype, diets containing ≥8% RS reduced adiposity compared to 0% RS. Energy intake decreased by 9.8 kJ/d for every 4% increase in RS. All diets containing RS increased total SCFA pools in the caecum and lowered plasma GIP concentrations compared to the 0% RS, whereas plasma GLP-1 and PYY were increased when the diet contained at least 8% RS. Insulin sensitivity was not affected by RS.

**Conclusion:**

RS in amounts that could be potentially consumed by humans were effective in reducing adiposity and weight gain in OP and OR rats, due in part to a reduction in energy intake, and changes in gut hormones and large bowel carbohydrate fermentation.

## Background

The global rise in Type 2 diabetes mellitus (T2DM) prevalence in industrialised countries poses a very serious public health problem through its attendant complications. In Australia, as in many other countries, health authorities are targeting primary and secondary prevention strategies, in particular dietary change, as a means of tackling the burgeoning T2DM epidemic [1]. As excess adiposity (especially abdominal) is a strong risk factor, approaches that reduce obesity are especially effective in lowering the prevalence of T2DM [2]. A dramatic reduction in risk of T2DM by at least 27% has been consistently shown when people consume higher levels of whole grain foods and cereal fibre [3]. Importantly, the data show that the metabolic health benefits are independent of reductions in body weight [3].

Resistant starch (RS) is the fraction of dietary starch that escapes digestion in the small intestine and passes into the large bowel of healthy humans thereby contributing to total dietary fibre intake [4]. The benefits of RS for gut health are well-established and are mediated largely through large bowel microbial fermentation products, specifically short chain fatty acid (SCFA) [4]. Evidence for a promising role for this particular type of fibre in the prevention and management of T2DM is growing. RS can act directly by reducing the glycaemic impact of a food by displacing digestible carbohydrate [5], but other mechanisms seem to also contribute. Recently, Aziz et al. [6] showed that in diet-induced obese rats, diets high in RS (as high-amylose maize starch; HAMS) reduced body weight gain (by 40%), fat pad weight and glycaemic response, and increased insulin sensitivity compared to a diet low in RS. Although these changes were dramatic they were achieved at levels of RS that are not readily achievable in the human diet. We have shown previously that moderate to high levels of RS (as 20% HAMS providing 6% RS) reduced the body weight of healthy non-obese rats compared to those fed a low-amylose control diet [7]. A study by Higgins et al. [8] also showed that a diet containing 13% HAMS reduced adiposity but not weight regain in diet-induced obese rats. Because dietary intakes of RS in industrialised countries are low, it is important to establish minimal levels of RS that elicit favourable metabolic effects in obese and non-obese animals to assist in identifying appropriate levels for human intervention trials.

This study aimed to determine whether there is a dose-dependency or threshold effect of RS intake (as HAMS) on body weight gain, adiposity and insulin sensitivity in both obesity prone (OP) and obesity resistant (OR) rats. The diet-induced obese rat model mimics closely the major changes seen in obese humans, in particular whereby only a subset of the animals develop insulin resistance and dyslipidemia [9]. The secondary aims were to determine if RS fermentation in the large bowel is associated with changes in plasma gut hormone levels and whether they correlate with adiposity.

## Methods

### Rats and diets

Nine wk old, male Sprague–Dawley rats (Mean ± SE, 319 ± 5 g, n=120), were obtained from the Animal Resource Centre, Western Australia. Rats were housed in groups of 3–4 rats in wire-bottomed cages in a room with controlled heating and lighting (23°C with a 12-h light/dark cycle) and had free access to food and water. After arrival, the rats were adapted to a non-purified commercial diet for 1 wk. All procedures involving animals were approved by the Commonwealth Scientific and Industrial Research Organisation Food and Nutritional Sciences Animal Ethics Committee.

#### Pre resistant starch intervention

The rats were provided with a modified AIN-93G diet [10] that contained a moderate amount of fat for 4 wk to induce obesity (Table 1). After 3 wk the 40% of rats that had gained the most weight were classified as OP and the 40% of rats that gained the least weight were classified as OR. After another week, 16 rats were randomly selected (Baseline group containing OP (*n*=8) and OR (*n*=8) rats) for baseline measurements of body composition and intestinal digesta. The remaining rats were allocated randomly to one of five dietary treatment groups containing equal numbers (*n*=8) of OP and OR rats.

**Table 1:** Composition of the diet (as fed)

	**Moderate fat**	**Resistant starch**				
		**0%**	**4%**	**8%**	**12%**	**16%**
Ingredients, g/kg						
LAMS ^1^	215	530	400	270	130	0
HAMS ^2^	0	0	130	260	400	530
Casein	190	200	200	200	200	200
Maltodextrin10	75	0	0	0	0	0
Sucrose	290	100	100	100	100	100
Anhydrous milk fat ^3^	44.2	0	0	0	0	0
Sunflower seed oil	118	70	70	70	70	70
Wheat bran	0	50	50	50	50	50
α-cellulose	30	0	0	0	0	0
Vitamins ^4^	11	10	10	10	10	10
Minerals ^4^	40	35	35	35	35	35
L-cystine	3	3	3	3	3	3
Choline bitartrate	2.5	2.5	2.5	2.5	2.5	2.5
Energy^5^, kJ/g	17.9	15.7	15.1	14.5	13.8	13.2

^1^ LAMS, low amylose maize starch.

^2^ HAMS, high amylose maize starch was 1043 (National Starch, Australia), type 2 RS (RS2), composition per 100 g; 91 g total starch, 30 g resistant starch, 10.8 g moisture, 0.2 g fat, 0.8 g protein.

^3^Anhydrous milk fat, 99.9% fat (Fonterra, Mount Waverley, Australia).

^4^ Vitamin and mineral mix AIN-93G [10].

^5^ Energy content of the diets was calculated based on energy values provided by the manufacturer for individual ingredients. An energy value of 10.45 kJ was used for HAMS [11], as referred to by Aziz et al. [6].

#### Resistant starch intervention

The diets were based on AIN-93G formulation [10] and contained increasing levels of HAMS (1043 National Starch, Sydney, Australia) a type 2 RS (RS2) (Table 1). The HAMS contained 91 g total starch, 8 g moisture, 0.2 g fat and 0.8 g protein per 100 g. A low-amylose maize starch (Avon maize starch, New Zealand Starch, Auckland, NZ) was used to balance starch levels in the diet and contained 87 g total starch, 0.7 g resistant starch, 11.9 g moisture, 0.1 g fat and 0.3 g protein per 100g. Rats had free access to the powdered diets which were fed for 4 wk. The amounts of RS in the diets as fed were 0, 4, 8, 12 and 16 g/100 g of diet. These levels are based on HAMS containing 30% RS as determined previously by us in pig and human studies [12,13].

At 0, 4 and 7 wk of the study the rats were deprived of feed overnight (12 h) and anaesthetised using 40 mg/kg Zoletil (Virvac, Sydney, Australia). Blood was taken from a tail vein to determine glucose and insulin concentrations and body composition analysis was conducted by dual energy X-ray absorptiometry (DXA); Lunar Prodigy with Encore 2007 software version 11.40.004 (GE Medical Systems, Madison, WI). A whole body scan was performed to measure body weight, bone mineral content, body fat and lean mass. Body weight, measured by DXA as the sum of lean mass, fat and bone mineral content, was 1.1 ± 0.1% (n=120) lower than body weight measured gravimetrically.

At 2 and 6 wk of the study, diet intake was measured by isolating rats in individual cages for 48 h. The amount of diet remaining at 24 and 48 h was weighed and average daily diet intake calculated. Average daily gross energy intake was calculated by multiplying the average daily diet intake by the energy density of the diet. The amount of RS in grams consumed by each rat per day for each diet was calculated by multiplying the average daily food intake (in g) for each rat by the amount of RS in the diet (in g per g). In the final week of the study faecal samples were collected from rats immediately after defecation and promptly frozen at −20°C for fat analysis.

At the conclusion of the study rats were anesthetised with 5% isoflurane in oxygen. Blood was collected from the abdominal aorta, processed after 30 min to obtain EDTA plasma and stored at −80°C until analysed. The major organs and adipose tissue, including mesenteric, epididymal, retroperitoneal and inguinal fat were removed and weighed. Visceral weight was reported as the sum of mesenteric, epididymal and retroperitoneal fat pad weights. The caecum and colon were weighed separately and their contents removed and weighed. The pH of the digesta was determined (Activon, Melbourne, Australia) and samples stored at −20°C for SCFA analysis. The weight of the full caecum was used to correct final body weight and weight gain, as described previously [6], in order to account for the increase in caecal tissue and digesta mass with increasing RS in the diet.

### Blood biochemistry

Plasma glucose, triglyceride and total cholesterol concentrations were measured using standard Roche enzymatic kits (Roche Diagnostics Co) and plasma non-esterified fatty acids (NEFA) were measured using a Randox kit. Assays were conducted using a BM/Hitachi 902 Automatic Analyzer. Fasting plasma insulin and non-fasted GLP-1 (total) were analysed by ELISA (Millipore).

The concentrations of insulin, GIP, PYY and leptin in non-fasted plasma collected at necropsy were determined using a rat gut hormone multiplex kit (Millipore, St. Charles, MO) according to manufacturer’s instructions. Multianalyte profiling was performed on the Qiagen LiquiChip 200 Workstation and fluorescence data were analysed by using the Qiagen LiquiChip Analyzer Software (version 1.0.5). A sub-set of samples from each dietary treatment group (n=8) were analysed in one run on one plate and the mean intra-assay variability ranged from 8 to 14%.

The homeostasis model assessment of insulin resistance (HOMA-IR) was calculated from fasting glucose and insulin levels and applying the following formula that has been validated for use in rats [14]. HOMA-IR = (fasting plasma glucose mg/dL x fasting plasma insulin μU/mL)/2,430).

### Digesta and liver analyses

Faecal and caecal contents were distilled and analysed for SCFA by gas chromatography, as described previously [15]. The total SCFA levels reported were the sum of the major (acetate, propionate and butyrate) and minor SCFA (isobutyric, isovaleric, valeric and caproic).

The fat content of liver and faeces was extracted by the modified method of Folch et al. [16]. In brief, approximately 1 g of liver tissue and 0.5 g faeces were freeze dried for 24 h then ground to a fine powder using a mortar and pestle. The powder was acidified with 25% HCl, homogenised and then extracted twice using 2:1 chloroform:methanol. The extracts were dried under nitrogen and weighed. Liver fat was expressed as a percentage of liver weight. Daily faecal fat excretion was calculated by multiplying the percentage of faecal fat by the daily faecal wet weight excreted in 24 h.

### Statistical analyses

The data are presented as the arithmetic mean and SEM for each treatment group. Growth rate, body composition data (as measured by DXA), and fasting glucose data obtained during the pre-RS treatment phase and the RS intervention were assessed using a repeated measures 2-way ANOVA. For the RS intervention, the data were analysed as a randomised complete block design with 2 x 5 factorial treatment structure using a 2-way ANOVA. Change in fat pad weights and total body fat mass were analysed using energy intake as a covariate. Significant interactions between RS and phenotype were analysed using pair-wise comparisons of simple main effects and applying a Bonferroni adjustment for multiple comparisons. In the absence of an interaction, difference between treatments was assessed by a Tukey’s post-hoc test. Standard multiple regression analysis was used to estimate correlations between variables and energy intake data were analysed by standard linear regression. These analyses were performed using SPSS version 18.0 (SPSS Inc., Chicago Il USA). A value of *P* < 0.05 was taken as the criterion of significance.

## Results

### Pre resistant starch intervention

Prior to commencing the moderate fat diet OP rats were heavier than OR rats (*P*<0.01) (Table 2). Thereafter, OP rats gained 7% more weight (*P*<0.0001) and 1.6% more total fat mass (*P*<0.01) in comparison to OR rats (Table 2). OP rats also consumed more food (OP; 22.2 ± 0.4, OR; 19.8 ± 0.3 g/d, *P*<0.0001) and energy per d (OP; 399 ± 7, OR; 355 ± 6 kJ/d, *P*=0.0001) in comparison to the OR rats. However, fasting blood glucose and insulin levels, and insulin resistance, were all similar for both OP and OR rats after consuming the moderate fat diet for 4 wk (Table 2).

**Table 2:** Change in body weight gain, body composition and serum biochemistry of obesity-prone and obesity-resistant rats after 4 wk on a moderate fat diet

	**Obesity resistant**			**Obesity prone**		
	**wk 0**^**1**^	**wk 4**^**1**^	**Δ (%)**^**2**^	**wk 0**^**1**^	**wk 4**^**1**^	**Δ (%)**^**2**^
Body weight, *g*	297 ± 4	366 ± 5	26	322 ± 4	427 ± 5	33 **
Fat mass, %	5.8 ± 0.3	10.5 ± 0.5	4.7	7.0 ± 0.3	13.3 ± 0.6	6.3 *
Bone mineral density, g/cm^3^	0.183 ± 0.001	0.207 ± 0.001	13.6	0.185 ± 0.001	0.216 ± 0.0001	17.4 **
Fasting glucose, mmol/L	5.3 ± 0.1	6.3± 0.1	20	5.5 ± 0.1	6.4 ± 0.1	18
Fasting insulin^3^, pmol/L	94 ± 27	267 ± 48	210	141 ± 32	344 ± 56	250
HOMA-IR^3^	0.5 ± 0.1	1.8 ± 0.3	300	0.9 ± 0.2	2.5 ± 0.5	280

^1^*n*=48 obesity-resistant, *n*=48 obesity-prone.

^2^ Percent change from 0 to 4 wk. Significant difference between obesity-resistant and obesity-prone rats is denoted by * *P* < 0.05, ** *P* < 0.0001.

^3^ Fasting insulin and HOMA-IR were measured in a subset of rats; *n*=4 obesity resistant and *n*=3 obesity prone.

### Resistant starch intervention

#### Diet, gross energy and RS intakes

The OP rats in comparison to OR rats consumed more food (OP; 25.5 ± 0.5, OR; 23.9 ± 0.3 g/d, *P*<0.05) and energy per d (OP; 368 ± 8, OR; 345 ± 6 kJ/d, *P*=0.05). The level of RS in the diet tended (*P*=0.076*)* to reduce energy intake (Table 3). However, linear regression analysis showed that for every 4% increase in the amount of RS in the diet, energy intake decreased by 9.8 kJ/d (*P* < 0.01). Average RS intakes were 0, 1.2, 2.5, 3.9 and 5.0 g/d for 0, 4, 8, 12 and 16% RS diets respectively.

**Table 3:** **Diet and energy intakes, body weight, liver weight and fat content of obesity-prone and obesity-resistant rats consuming diets with different levels of resistant starch**^**1**^

	**Resistant starch (RS), % of diet weight**					**Main Effects (*P*-value)**		
	**0%**	**4%**	**8%**	**12%**	**16%**	**RS**	**Phenotype**	**RS x Phenotype**
Gross energy intake^2,3^, *kJ/d*	378 ± 12	364 ± 7	355 ± 12	345 ± 12	338 ± 10	0.076	0.018^7^	0.857
Final body weight^2,4^, *g*	485 ± 15	465 ± 12	445 ± 18	474 ± 11	461 ± 14	0.136	0.0001^8^	0.692
Final body weight corrected^2,4,5^, *g*	483 ± 15	462 ± 12	437 ± 18	467 ± 12	449 ± 13	0.06	0.0001^9^	0.724
Liver wt^2,4^, *%BW*	3.19 ± 0.08 ^c^	3.15 ± 0.03 ^bc^	3.02 ± 0.05 ^ab^	3.04 ± 0.05 ^bc^	2.83 ± 0.07 ^a^	0.0001	0.126	0.056
Liver fat content^6^, *%*	6.4 ± 0.4	6.4 ± 0.3	6.3 ± 0.1	6.5 ± 0.4	6.6 ± 0.4	0.764	0.010^10^	0.613

^1^ Values are means ± SEM. Labelled means without a common letter differ, *P* < 0.05.

^2^*n*=8 obesity-prone and *n*=8 obesity-resistant rats.

^3^ Measured after rats were fed the RS diets for 2 wk.

^4^ Measured after rats were fed the RS diets for 4 wk.

^5^ Final body weight corrected = body weight – full caecum weight.

^6^ n=8 (n=4 obesity-prone and n=4 obesity-resistant rats per group).

^7^ Obesity-prone 368 ± 8 kJ/d, Obesity-resistant 345 ± 6 kJ/d.

^8^ Obesity-prone 502 ± 7 g, Obesity-resistant 445 ± 6 g.

^9^ Obesity-prone 497 ± 7 g, Obesity-resistant 441 ± 6 g.

^10^ Obesity-prone 7.0 ± 0.2%, Obesity-resistant 6.1 ± 0.2%.

#### Body weight and composition

**Figure 1:**
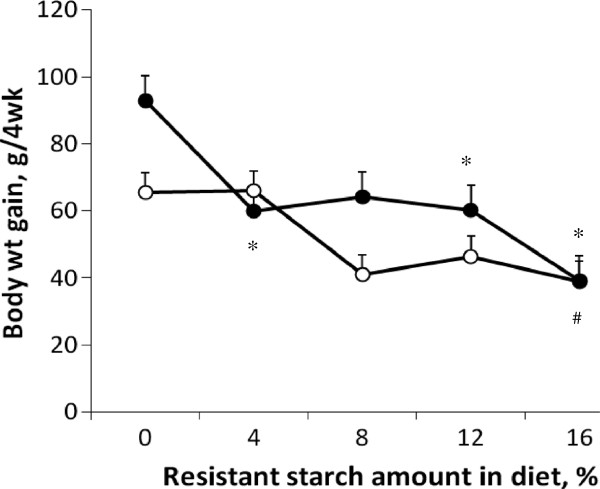
**Body weight gain in obesity-prone (●) and obesity-resistant (○) Sprague Dawley rats fed differing levels of resistant starch (RS) for 4 wk.** Body weight gain is corrected body weight as calculated by the following formula; (final body weight – full caecum weight) – initial body weight. Values are expressed as mean ± SEM. There was a significant interaction between RS and phenotype (*P* < 0.05). A significant difference in comparison to 0% RS is denoted by (*) for obesity prone rats and (#) for obesity resistant rats, *P* < 0.05. Dietary treatment groups were comprised of a total of 16 animals; *n*=8 obesity prone and *n*=8 obesity resistant.

The effect of RS on body weight gain was dependent on phenotype (*P*<0.05) (Figure 1). For OP rats, the addition of 4%, 12% or 16% RS to the diet reduced weight gain in comparison to the 0% RS diet, whereas in OR rats, weight gain was only reduced by the 16% RS diet (Figure 1). RS did not affect final body weight independent of the obesity phenotype (Table 3). However, there was a trend for RS to lower final body weight corrected for caecum full weight (P=0.06) (Table 3). Final body weight and corrected final body weight were higher for the OP animals in comparison to OR animals (Table 3).

**Figure 2:**
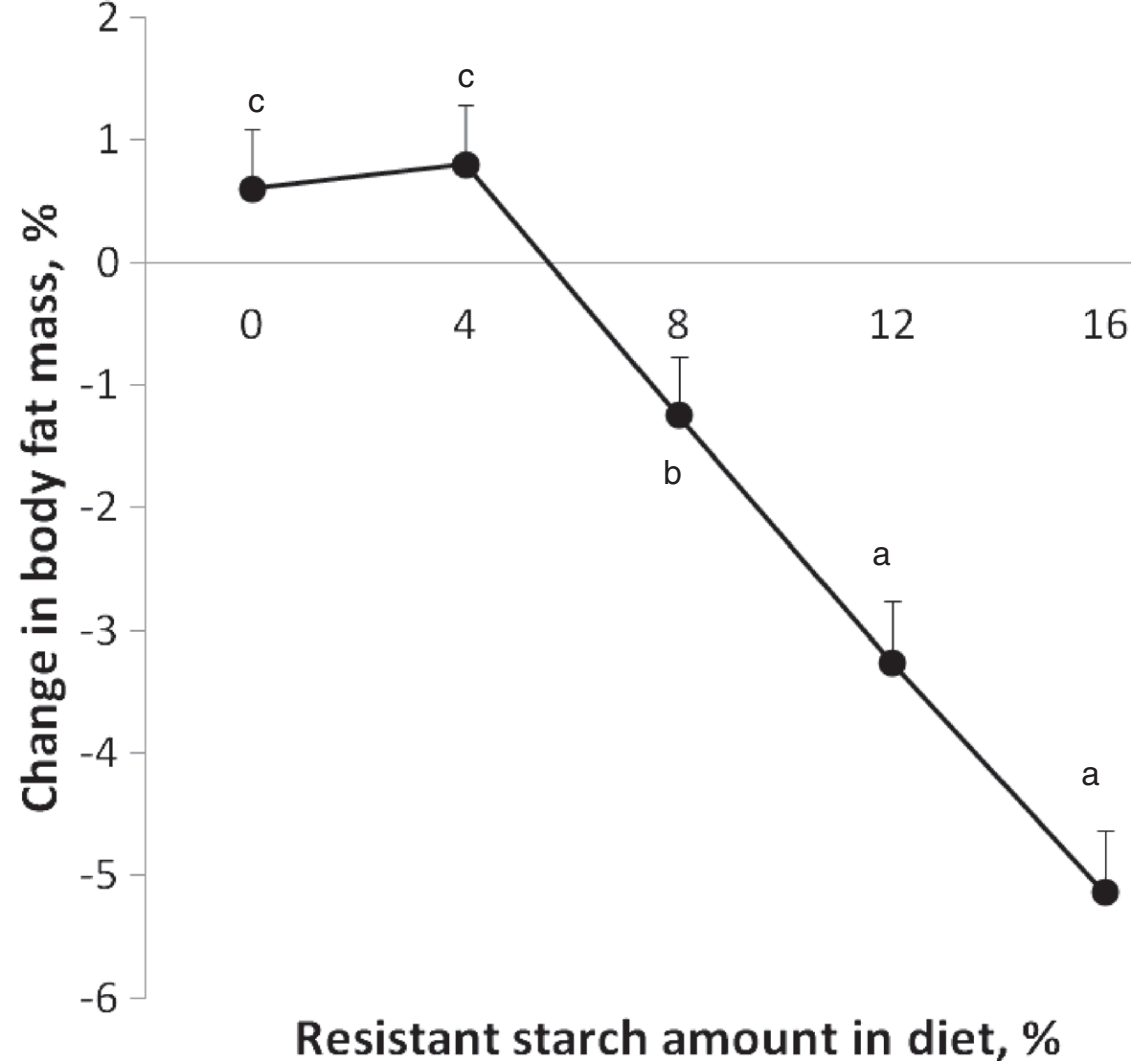
**Change in body fat mass in Sprague Dawley rats fed differing levels of RS for 3 wk.** Values are expressed as mean ± SEM. Change in body fat mass was affected by RS (*P* <0.0001). Dietary treatment groups were comprised of a total of 16 animals; *n*=8 obesity-prone and *n*=8 obesity-resistant.

**Figure 3:**
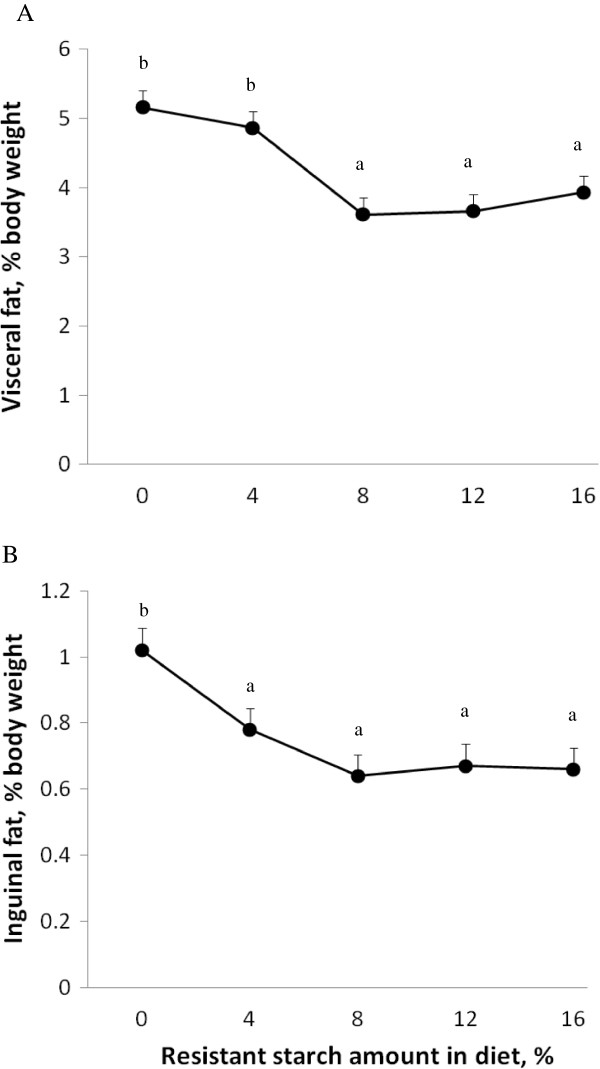
**Visceral fat mass (A) and inguinal fat mass (B) in Sprague Dawley rats fed differing levels of resistant starch (RS) for 4 wk.** Values are expressed as mean ± SEM. Visceral fat mass was the sum of mesenteric, epididymal and retroperitoneal fat pad weights and was affected by RS (*P* <0.0001) and phenotype (*P* <0.0001). Inguinal fat mass was only affected by RS (*P* <0.0001). *Dietary* treatment groups were comprised of a total of 16 animals; *n*=8 obesity prone and *n*=8 obesity resistant.

The RS diets lowered adiposity regardless of whether animals were OP or OR. After 3 wk, a minimum of 8% RS reduced total body fat in comparison to the 0% RS fed rats (Figure 2). At 4 wk, there was less visceral fat in rats on diets containing 8% or more RS (Figure 3). This reduction in visceral fat mass was similar for all visceral fat sites including mesenteric fat, epididymal fat and retroperitoneal fat (data not shown). Visceral fat mass was also greater in OP (4.7 ± 0.2% body weight) than OR rats (3.8 ± 0.1% body weight) (*P*<0.0001). Inguinal fat mass was reduced by diets containing 4% or higher RS in comparison to the 0% RS group (Figure 3). In addition, these effects of RS on reducing total body fat, and visceral and subcutaneous fat depots were maintained even when energy intake was included as a covariate.

After 4 wk on the RS diets, rats fed 8% or more RS had visceral and subcutaneous fat contents similar to the baseline rats (visceral fat, 3.9 ± 0.2% body weight and subcutaneous fat 0.7 ± 0.06% body weight). In comparison to the baseline group, the 0 and 4% RS group accumulated more visceral (0% RS, 62%, *P*<0.0001; 4% RS, 37%, *P*<0.01) and subcutaneous fat (0% RS, 67%, *P*<0.0001, 4% RS, 19%, *P*<0.0001).

Lean tissue as a percentage of body weight was not affected by the amount of RS added to the diets (data not shown). Lean tissue of OR (11.8 ± 0.7%) and OP rats (12.3 ± 0.7%) increased by similar amounts during the RS intervention.

RS affected liver weight, independent of obesity phenotype. Liver weights of rats fed 8% (*P*<0.05) and 16% RS (*P*<0.0001) were lighter than those of the 0% RS group (Table 3). Hepatic lipid content was higher for OP rats in comparison to OR rats, but was not affected by the amount of RS in the diet (Table 3). However, the livers of rats fed ≥ 0% RS tended to contain less lipid (6.5 ± 0.2%) than the baseline group (7.6 ± 0.3%) (*P*=0.06). RS content of the diet did not affect heart weight, spleen weight or kidney weight (data not shown).

#### Large bowel variables

The 4% RS group in comparison to the 0% RS group increased colonic digesta weight, total SCFA pools and pH (Table 4). In addition, the inclusion of a minimum of 8% RS in the diet increased a number of general bowel health endpoints, including caecal total SCFA pools, caecal digesta weight and faecal output in comparison to the 0% RS diet (Table 4). The changes in caecal digesta weight in response to the level of RS in the diet were also dependent on phenotype. In OR rats caecal digesta weight reached a maximal level with 12% RS whereas in OP rats caecal digesta weight was maximal at the highest level of RS (16%) (Table 4).

**Table 4:** **Large intestinal tissue weights and fermentation parameters of obesity-prone and obesity-resistant rats consuming diets with different levels of resistant starch for 4 wk**^**1**^

	**Resistant starch, % of diet weight**					**Main Effects (*P*-value)**		
	**0%**	**4%**	**8%**	**12%**	**16%**	**RS**	**Phenotype**	**RS x Phenotype**
**Caecum**								
Digesta weight, *g*								0.015
Obesity-prone	1.7 ± 0.2 ^a^	2.3 ± 0.3 ^a^	6.4 ± 1.0 ^b^	6.9 ± 1.0 ^b^	11.5 ± 1.6 ^c^			
Obesity-resistant	1.5 ± 0.1 ^a^	2.5 ± 0.5 ^ab^	6.0 ± 0.7 ^b^	9.5 ± 0.9 ^c^	7.4 ± 1.3 ^bc^			
Total SCFA pool, *mmol*	169 ± 11 ^a^	288 ± 43 ^ab^	657 ± 83 ^bc^	985 ± 150 ^c^	1029 ± 150 ^c^	0.0001	ns	ns
Digesta pH	7.2 ± 0.1 ^c^	6.8 ± 0.1 ^b^	6.2 ± 0.1 ^a^	6.0 ± 0.1 ^a^	5.9 ± 0.1 ^a^	0.0001	ns	ns
**Colon**								
Digesta weight, *g*	1.0 ± 0.1 ^a^	1.9 ± 0.1 ^b^	2.1 ± 0.2 ^bc^	3.0 ± 0.2 ^d^	3.2 ± 0.3 ^d^	0.0001	ns	ns
Total SCFA pool, *mmol*	69 ± 7 ^a^	160 ± 14 ^b^	184 ± 26 ^b^	213 ± 20 ^b^	215 ± 23 ^b^	0.0001	0.016^3^	ns
Digesta pH	7.3 ± 0.1 ^c^	6.3 ± 0.1 ^b^	6.0 ± 0.1 ^ab^	6.0 ± 0.1 ^ab^	5.8 ± 0.1 ^a^	0.0001	ns	ns
**Faeces**								
Faecal output *g/d*	1.7 ± 0.1 ^a^	3.0 ± 0.2 ^ab^	3.7 ± 0.3 ^b^	5.8 ± 0.6 ^c^	8.3 ± 0.5 ^d^	0.0001	0.028^4^	ns
Faecal fat^2^, *g/d*	0.15 ± 0.02	0.09 ± 0.01	0.11 ± 0.02	0.09 ± 0.01	0.13 ± 0.01	ns	ns	ns

^1^ Values are means ± SEM. Labelled means without a common letter differ, *P* < 0.05. ns = not significant, *P* > 0.05. All dietary treatments consist of *n*=8 obesity-prone and *n*=12 obesity-resistant rats.

^2^*n*=8 (*n*=4 obesity-prone and *n*=4 obesity-resistant rats per group).

^3^ obesity-prone 188 ± 11 mmol, obesity-resistant 149 ± 11 mmol.

^4^ obesity-prone 4.9 ± 0.5 g/d, obesity-resistant 4.3 ± 0.2.

The amount of fat excreted in faeces was not affected by the addition of RS to the diet or the obesity phenotype (Table 4).

#### Insulin sensitivity, lipids and gut hormones

The amount of RS in the diet or obesity phenotype did not affect fasting plasma levels of glucose or insulin, or insulin resistance as determined by HOMA-IR ( Additional file 1: Table S1).

The inclusion of 8% RS or more in the diet reduced plasma triglyceride and total cholesterol concentrations in comparison to the 0% RS group (Table 5). Plasma leptin concentration was higher in OP than OR rats (P < 0.05) and was reduced when the diet contained 8% and 16% RS (P < 0.05) (Table 5). The addition of RS to the diets did not affect plasma NEFA concentration.

**Table 5:** **Plasma lipids and gut hormones of obesity-prone and obesity-resistant rats after consuming diets with different levels of resistant starch for 4 wk**^**1**^

	**Resistant starch (RS), % of diet weight**					**Main Effects (*P*-value)**		
	**0%**	**4%**	**8%**	**12%**	**16%**	**RS**	**Phenotype**	**RS x Phenotype**
Triglycerides^2^, *mmol/L*	1.4 ± 0.1 ^c^	1.1 ± 0.1 ^bc^	0.8 ± 0.1 ^ab^	0.9 ± 0.1 ^ab^	0.7 ± 0.1 ^a^	0.0001	ns	ns
Free fatty acids^2^, *mmol/L*	0.30 ± 0.02	0.29 ± 0.02	0.32 ± 0.03	0.34 ± 0.04	0.30 ± 0.03	ns	ns	ns
Total cholesterol^2^, *mmol/L*	1.8 ± 0.1 ^c^	1.8± 0.1 ^bc^	1.6 ± 0.1 ^ab^	1.5 ± 0.1 ^ab^	1.5 ± 0.1 ^a^	0.002	ns	ns
Leptin^3^, *μg/L*	14.7 ± 4.7 ^b^	11.7 ± 1.1 ^ab^	7.3 ± 4.5 ^a^	8.3 ± 0.9 ^ab^	5.3 ± 0.8 ^a^	0.027	0.042^4^	ns
GLP-1^3^, *pg/mL*	29 ± 4 ^a^	47 ± 4 ^ab^	70 ± 7 ^bc^	76 ± 6 ^bc^	95 ± 23 ^c^	0.0001	ns	ns
PYY^3^, *pg/mL*	71 ± 14 ^a^	137 ± 11 ^ab^	230 ± 30 ^bc^	222 ± 22 ^bc^	297 ± 54 ^c^	0.0001	ns	ns
GIP^3^, *pg/mL*	372 ± 57 ^a^	165 ± 29 ^b^	134 ± 25 ^b^	176 ± 52 ^b^	81 ± 18 ^b^	0.001	ns	ns

^1^ Values are means ± SEM. Labelled means without a common letter differ, *P* < 0.05. ns = not significant, *P* > 0.05. GLP-1; glucagon-like peptide −1, PYY; polypeptide Y, GIP; gastric inhibitory peptide.

^2^*n*=16 (Obesity-prone; *n*=8, Obesity-resistant; *n*=8).

^3^*n*=8 (Obesity-prone; *n*=4, Obesity-resistant; *n*=4).

^4^ obesity-prone 11.7 ± 1.7 μg/L, obesity-resistant 7.3 ± 1.1 μg/L.

Plasma gut hormone concentrations were affected by the level of RS in the diet, but unaffected by phenotype (Table 5). GLP-1 and PYY increased when a minimum of 8% RS was included in the diet (*P* < 0.05) whereas GIP concentration was lower for all diets containing added RS (*P* < 0.05) (Table 5). Caecal digesta SCFA pools were associated with concentrations of plasma GLP-1 (r=0.317, n=38, *P* < 0.005) and PYY (r=0.433, both n=38, *P* < 0.005) and inversely associated with plasma GIP (r=−0.538, n=37, *P* < 0.001). Plasma GLP-1 and PYY were inversely associated with total body fat mass (GLP-1; r=−0.396, n=39, P<0.01, PYY; r=−0.421, n=39, *P* < 0.01) and visceral fat mass (GLP-1; r=−0.428, n=39, *P* < 0.01, PYY; r=−0.438, n=39, *P* < 0.01). PYY was inversely associated with subcutaneous fat mass (r=−0.308, n=38, *P* < 0.036), but GLP-1 was not (r=−0.224, n=36, *P* = 0.098).

## Discussion

Animal studies consistently show that diets containing high levels of RS (16%) reduce body weight gain and/or adiposity in rodents [6,17,18]. Moderate levels of RS (4-6%) also reduced weight gain in healthy rats [7] and adiposity during weight regain in obese rats [8]. However, the effects of moderate to high RS levels in the diet on adiposity and weight gain have not been examined in OP or OR rodents without prior exposure to a weight loss diet. The present study demonstrated clearly that dietary RS reduced body weight gain, although not final body weight, and that the effect was dependent on the level of RS in the diet and on the phenotype of the rats. When compared to 0% RS, weight gain was significantly lower in OP rats when fed RS at 4, 12 and 16% whereas in OR rats, weight gain was significantly lower only when fed 16% RS. These differences between obesity phenotype could be due to differences in colonic fermentation. In support of this Zhou et al. [18] demonstrated that RS did not reduce adiposity in genetically obese mice that were unable to ferment RS. Additionally studies by Gordon and colleagues show that differences in microbial populations between obese and lean individuals may explain the phenotypic differences [19] however differences in energy intake may also account for this effect. The current study demonstrated that the level of RS in the diet necessary to reduce adiposity, was independent of the obesity phenotype. Although OP rats fed 4% RS gained less weight than rats on the 0% RS diet, visceral and subcutaneous adipose tissue weights, total adiposity and energy intake did not differ. In humans replacement of 5.4% of total dietary carbohydrate with RS (approximately 5 g RS in the meal) increased postprandial lipid oxidation [20], however consumption of RS (40 g/d) for 12 wk did not affect body weight or fat storage in muscle, liver and visceral depots in overweight/obese subjects [21]. The higher dietary levels of RS (8%) used in the present study lowered total body and visceral adipose tissue weight, as well as plasma triglycerides, total cholesterol and leptin. This level of RS intake, which equates to about 88 g/d for a 75 kg human, would be difficult to achieve for most adults [22]. However it is possible that lower amounts (between 44 and 88 g/d for an adult) that could be readily accommodated in the diet via dietary supplements and consumption of foods high in RS, may elicit favourable changes in adiposity. Furthermore, at these lower levels of RS a greater reduction in adiposity may also be achieved in a trial of longer duration.

The mechanisms by which RS reduces adiposity are likely to involve a decrease in metabolisable energy intake and an increase in fatty acid catabolism. In the current study gross energy intake declined as the amount of RS increased in the diet (16% RS reduced energy intake by 11%). An even larger reduction in energy intake (16.4%) was reported for rats consuming a high RS diet (equivalent to 16% RS) [6] when total accumulative energy intake was measured daily throughout the 4 wk trial rather than a 2-day measurement period used in the current study. In addition, studies that matched the energy content of the control (0% RS) and RS intervention diets, still resulted in a more effective lowering of body fat than the control [17,18] which suggests that mechanisms other than reducing energy intake may also play a role in reducing adiposity. Fermentation of RS in the large bowel has been proposed as a mechanism to explain the increase in lipid oxidation [18,23]. SCFA, the major products of RS fermentation, are absorbed by colonocytes and enter the hepatic portal circulation where they can directly regulate a variety of pathways involved in fatty acid and cholesterol metabolism [24-26]. In particular, propionate can reduce the incorporation of acetate into cholesterol and inhibits fatty acid synthesis [27,28]. Additionally, a lower ratio of serum acetate to propionate is associated with lower serum cholesterol [29,30] but was not seen in the current study. Although most SCFA produced in the colon are metabolised by the liver small amounts enter the systemic circulation [31,32] and there is growing evidence that they may directly regulate adipogenesis and adipokine release in adipose tissue mediated via G-protein coupled receptors [25]. Fermentation of a single meal high in RS (measured using breath hydrogen) was associated with a reduction in plasma NEFA levels in healthy subjects [33]. This reduction in NEFA may be particularly effective in improving insulin sensitivity in obese individuals and those with T2DM as it would reduce fatty acid oxidation and storage, increase muscle glucose uptake and oxidation, and improve β-cell insulin secretory response to glucose [28]. However, in the current study it is not clear why RS did not reduce plasma NEFA, but it may explain in part why an improvement in insulin sensitivity was not seen. Additionally, SCFA may modulate fat metabolism indirectly by stimulating large bowel enteroendocrine cell production of GLP-1 and PYY [29]. In the current study total SCFA pools in caecal digesta were positively associated with plasma concentrations of both hormones. GLP-1 and PYY have been shown to be released into the blood in a sustained day-long manner [30] and to act systemically on white adipose tissue to regulate lipogenesis, lipolysis, fatty acid release and adipocyte differentiation [31]. Additionally, PYY can increase thermogenesis and energy expenditure, thereby reducing adiposity [32]. In support of this, plasma GLP-1 and PYY levels explained 16 – 19% of the change in total body fat mass and visceral fat mass. Furthermore, diets containing added RS reduced plasma concentrations of GIP, a gut hormone known to promote lipogenesis by stimulating adipose tissue blood flow, glucose uptake and fatty acid re-esterification, and, as a consequence, increased triglyceride deposition in abdominal subcutaneous adipose tissue [33,34].

Foods containing RS elicit lower postprandial insulin responses [34] and short-term consumption of RS (15–40 g/d) by healthy as well as overweight and obese subjects improves insulin sensitivity [21,35,36]. Studies in rats also show consistent positive effects for RS on postprandial glycemic and insulinemic responses and whole body insulin sensitivity [37]. Therefore, it was surprising in the current study that resistant starch even at the highest level of dietary inclusion did not improve insulin sensitivity of the rats. It is possible that the duration of the moderate fat diet was too short or that fasting levels of glucose and insulin were not sensitive enough to discern changes in insulin sensitivity. Higgins et al. [38] showed that rats fed an amylopectin-based diet (low in RS) developed insulin resistance (as measured by an intravenous glucose tolerance test) after 3 mo of feeding whereas those on an amylose diet (high in RS) developed insulin resistance after 6 mo. A feeding trial considerably longer than the 4 wk used in the current study may be required to investigate the dose response effects of resistant starch on improving insulin sensitivity.

The low fat background diet used in the present study is not representative of the typical Western diet and may have limited the impact of RS in improving insulin sensitivity. Andersson et al. [39] showed that a low dietary fat content ameliorated the negative effect of a high glycaemic diet on insulin resistance. In our study the low level of dietary fat was more effective in lowering hepatic fat levels than feeding RS. However the length of the intervention was only 4 wk and may have been too short to observe an effect of RS on reducing heptic lipid content.

## Conclusions

In summary, intakes of RS at levels that could be achieved by humans were effective in reducing adiposity and weight gain in rats, and also tended to reduce final body weight. At the lower levels of dietary RS (4%) OP rats gained less body weight in comparison to rats fed 0% RS, whereas for OR rats a higher level of RS (16%) was required to limit weight gain. This difference between phenotype is likely explained by differences in colonic fermentation and deserves further investigation. A role for RS fermentation in reducing adiposity is supported by the positive relationships between caecal digesta total SCFA pools and plasma gut hormones (GLP-1 and PYY) that were inversely associated with total body fat and visceral fat mass. Although OP rats did not develop insulin resistance, the lowering of plasma GIP by all dietary levels of RS was considerable and deserves further investigation in animal models with impaired insulin sensitivity.

## Abbreviations

DXA: Dual-energy X-ray absorptiometry; GIP: Gastric inhibitory peptide; GLP-1: Glucagon-like peptide-1; HOMA-IR: Homeostasis model assessment of insulin resistance; NEFA: Non-esterified fatty acids; OP: Obesity-prone; OR: Obesity-resistant; PYY: Polypeptide-Y; RS: Resistant starch; SCFA: Short chain fatty acid; T2DM: Type-2 diabetes mellitus.

## Competing interests

The authors declare that they have no competing interests.

## Authors’ contributions

D.P.B. conducted the study and analysed the data. All authors contributed to research design, data interpretation, manuscript preparation, and read and approved the final manuscript. A.R.B. had primary responsibility for final content.

## Funding

Financial support was provided by the CSIRO Food Futures Flagship.

## Supplementary Material

Additional file 1 **Table S1.** Plasma glucose, insulin and insulin sensitivity in feed-deprived obesity prone and obesity resistant rats after consuming diets with different levels of resistant starch for 3 wk^1^. Click here for file
