# Supplementary material for: Dietary resistant starch dose-dependently reduces adiposity in obesity-prone and obesity-resistant male rats
Source: Nutr Metab (Lond). 2012 Oct 25;9:93. doi: 10.1186/1743-7075-9-93 (PMC3541085; doi:10.1186/1743-7075-9-93)
Supplement: Additional file 1 — Table S1. Plasma glucose, insulin and insulin sensitivity in feed-deprived obesity prone and obesity resistant rats after consuming diets with different levels of resistant starch for 3 wk1. [file 1743-7075-9-93-S1.docx]

Supplemental Table 1. Plasma glucose, insulin and insulin sensitivity in feed-deprived obesity prone and obesity resistant rats after consuming diets with different levels of resistant starch for 3 wk^1^

|  | Resistant starch (RS) | | | | |  | Main Effects (*P*-value) | | |
| --- | --- | --- | --- | --- | --- | --- | --- | --- | --- |
|  | 0% | 4% | 8% | 12% | 16% |  | RS | Phenotype | RS x Phenotype |
| Glucose, *mmol/L* | 6.4 ± 0.2 | 5.9 ± 0.2 | 6.0 ± 0.2 | 6.3 ± 0.2 | 6.3 ± 0.2 |  | ns | ns | ns |
| Insulin, *pmol/L* | 241 ± 52 | 275 ± 52 | 172 ± 52 | 224 ± 52 | 293 ± 52 |  | ns | ns | ns |
| HOMA-IR^2^ | 1.6 ± 0.4 | 1.8 ± 0.4 | 1.1 ± 0.4 | 1.5 ± 0.4 | 1.9 ± 0.4 |  | ns | ns | ns |

^1^ Values are means ± SEM. ns= not significant, *P*>0.05. *n*=8 (Obesity prone; *n*=4, Obesity resistant; *n*=4)

^2^ HOMA-IR; homeostasis model assessment of insulin resistance
